# Association of Polymorphisms in *Toll-Like Receptors 4* and *9* with Autoimmune Thyroid Disease in Korean Pediatric Patients

**DOI:** 10.1155/2017/2304218

**Published:** 2017-08-20

**Authors:** Won Kyoung Cho, Jung-Pil Jang, Eun-Jeong Choi, Moonbae Ahn, Shin Hee Kim, Kyoung Soon Cho, So Hyun Park, In Cheol Baek, Min Ho Jung, Tai-Gyu Kim, Byung-Kyu Suh

**Affiliations:** ^1^Department of Pediatrics, College of Medicine, St. Vincent's Hospital, The Catholic University of Korea, Seoul, Republic of Korea; ^2^Department of Microbiology, College of Medicine, The Catholic University of Korea, Seoul, Republic of Korea; ^3^Catholic Hematopoietic Stem Cell Bank, College of Medicine, The Catholic University of Korea, Seoul, Republic of Korea; ^4^Department of Pediatrics, College of Medicine, Seoul St. Mary's Hospital, The Catholic University of Korea, Seoul, Republic of Korea; ^5^Department of Pediatrics, College of Medicine, Incheon St. Mary's Hospital, The Catholic University of Korea, Seoul, Republic of Korea; ^6^Department of Pediatrics, College of Medicine, Bucheon St. Mary's Hospital, The Catholic University of Korea, Seoul, Republic of Korea; ^7^Department of Pediatrics, College of Medicine, Yeouido St. Mary's Hospital, The Catholic University of Korea, Seoul, Republic of Korea

## Abstract

**Background:**

*Toll-like receptors* (*TLRs*) have been suggested to be associated with the development of AITD.

**Methods:**

Fifteen single-nucleotide polymorphisms in 7 *TLR* genes were analyzed in 104 Korean children (girls = 86, boys = 18) with AITD (Hashimoto disease (HD) = 44, Graves' disease (GD) = 60, thyroid-associated ophthalmopathy (TAO) = 29, and non-TAO = 31) with 183 controls.

**Results:**

GD showed higher frequencies of the *TLR4 rs1927911* C allele than control. TAO showed a lower frequency of the *TLR4 rs1927911* CT genotype and non-TAO showed a higher frequency of the *TLR4 rs1927911* CC genotype than control. The frequency of the *TLR9 rs187084* CC genotype in TAO was higher than that in non-TAO. GD females showed a higher frequency of the *TLR*4 *rs10759932* T allele, *rs1927911* CC genotype, and the *rs1927911* C allele than controls. GD males showed a higher frequency of the *TLR4 rs10759932* CC genotype and *rs1927911* TT genotype and lower frequency of the *rs1927911* CT genotype than control. The frequency of the *TLR4 rs10759932* CC genotype, C allele and *rs1927911* TT genotype, and T allele in a GD female were lower than in a GD male.

**Conclusions:**

Our results suggest that *TLR4* and *9* polymorphisms might contribute to the pathogenesis of GD and TAO.

## 1. Introduction

It has been suggested that autoimmune thyroid disease (AITD) may occur when genetically susceptible individuals are exposed to environmental triggers such as infection, iodine, and stress [1]. AITD are female predominant and the biology of sexual dimorphism in AITD is not clearly understood. Recently, much attention and research funding has been focused on gender-based differences in AITD. In Taiwan, nationwide cohort studies have reported that AITD, including Hashimoto disease (HD) and Graves' disease (GD), might be risk factors of developing thyroid, breast, and colon cancers later in life [2, 3].

*Toll-like receptors* (*TLRs*) recognize a variety of pathogen-associated molecular patterns such as bacteria, viruses, fungi, and certain host-derived molecules [4]. *TLRs* enable the innate immune system and induce a cascade of effector responses. *TLRs* are type I transmembrane glycoproteins with an extracellular domain of numerous leucine-rich repeats and an intracellular region containing a Toll *IL-1* receptor homology domain [5].

Previous disease association studies revealed the effect of *TLRs* on the development of chronic inflammatory disease, injury, and cancer [6]. *TLRs* including *TLR3* and *4* have been described on thyrocytes and reported to be associated with AITD or inflammatory disease [7–9]. In murine macrophages, gender difference in the expression of *TLR4* for bacterial LPS has been reported [10]. We have reported an association between *TLR10* polymorphisms and AITD [11]. In addition, *TLR9* polymorphisms have been reported to be associated with TAO in Taiwanese males [12].

Genetic susceptibility might be a greater concern in early onset of AITD than in late onset of the disease. In our previous study, we observed increased allele frequencies for *HLA-B^∗^46*, *HLA-DRB1^∗^08*, and *HLA-Cw^∗^01* in children with AITD than in the control group [13]. The statistical significance in our results were significantly stronger than any other study conducted on Korean adults [14]. The aforementioned strong statistical significance might suggest that early-onset AITD is more influenced by genetic factors than in late-onset cases. In this study, we investigated the potential associations of seven *TLR* genes (*TLR1*, *2*, *3*, *4*, *5*, *6*, and *9*) including 15 single-nucleotide polymorphisms (SNP) with AITD in Korean children. We also comprehensively analyzed the association of *TLR* genes with disease subgroups based on sex and thyroid-associated ophthalmopathy (TAO) of AITD.

## 2. Subjects and Methods

### 2.1. Participants

This study analyzed 104 patients diagnosed with AITD: 44 with HD and 60 with GD (TAO = 29, non-TAO = 31), who were treated at pediatric endocrine clinics at Seoul St. Mary's Hospital between March 2009 and August 2014. Of these patients, 84 were in a previous study conducted by our research group [11]. The age of patients at study enrollment was 13.2 ± 3.5 years and the age at AITD diagnosis was 11.3 ± 3.2 years (Table 1).

The control group consisted of 183 healthy Korean adults without a history of AITD, who were staff members and students at the College of Medicine at the Catholic University of Korea. All participants provided informed consent for a genetic study. This study was approved by the Institutional Review Board (IRB) of The Catholic University of Korea (IRB number: KC09FISI0042).

HD was diagnosed when at least three of the following criteria established by Fisher et al. [15] were met: goiter, diffuse goiter and decreased radionuclide uptake during thyroid scan, circulating thyroglobulin or microsomal autoantibodies, and hormonal evidence of hypothyroidism. GD was diagnosed based on clinical symptoms and biochemical confirmation of hyperthyroidism, including diagnosis of goiter, elevated radioactive iodine uptake, antibodies against the TSH receptor, and elevated thyroid hormone levels. Patients with other forms of autoimmune, hematologic, or endocrine diseases were excluded. TAO was diagnosed based on the presence of typical clinical features and classified according to the system recommended by the American Thyroid Association Committee [16, 17]. Patients with no symptoms or only a lid lag sign were included in the without-TAO group. Patients with soft tissue changes, proptosis, extraocular muscle dysfunction, or the latter two symptoms were considered to have an eye disease [18].

### 2.2. DNA Extraction

Genomic DNA was extracted from peripheral blood cells using AccuPrep Genomic DNA Extraction kits (Bioneer Corporation, Daejeon, Korea), according to the manufacturer's guidelines. The concentration of DNA solutions was adjusted to 100 ng/*μ*l and used as polymerase chain reaction (PCR) templates for genotyping.

#### 2.2.1. Analysis of TLR Polymorphisms

Genotyping was performed with using a direct sequence method. Fifteen SNPs of the 7 Toll-like receptor gene (*TLR1*, *2*, *3*, *4*, *5*, *6*, and *9*) were amplified by PCR using specific primers (Table 2). TLR4 has been described as a highly polymorphic gene [19]. The criteria the authors used to select the polymorphisms to be evaluated are as follows: First, genomic information of *TLR4* was investigated (https://www.ncbi.nlm.nih.gov/gene/7099). Based on the aforementioned investigation, we reviewed articles on disease associations with *TLR4* SNP. Among candidate's polymorphisms to be evaluated, we excluded polymorphisms having MAF 1.0 in population diversity of Japanese in Tokyo (https://www.ncbi.nlm.nih.gov/variation/tools/1000genomes/). In this process, some polymorphisms including *TLR4 rs4986791*(C = 1.000) and *rs4986790* (A = 1.000) were ruled out. Finally, the disease associations of *TLR4 rs1927911*, *rs10759932*, and *rs11536889* were evaluated. Other TLR genes were also determined in this way.

Amplification was performed in a GeneAmp PCR System 9700 thermocycler (Applied Biosystems, Foster City, CA, USA) using the following conditions: one cycle at 95°C for 5 min and 35 cycles of denaturation at 95°C for 30 sec, annealing at 55–62°C (depending on primer set) for 30 sec, extension at 72°C for 1 min, and final extension at 72°C for 10 min. The length of amplified products was confirmed by electrophoresis on 1.5% agarose gels.

PCR products from the second round were cleaned using exonuclease I and shrimp alkaline phosphatase (United States Biochemical) and used as sequencing templates. Sequencing was performed using a Big Dye Terminator version 3.1 (Amersham Pharmacia) and reactions were analyzed with ABI PRISM 3730XL analyzer (PE Applied Biosystems, Foster City, CA, USA). Sequencing data was analyzed with FinchTV software version 1.4 (Geospiza Inc., Seattle, Washington, USA).

#### 2.2.2. Statistical Analysis

Allele frequencies were determined using Microsoft Office Excel. For controls, Hardy-Weinberg equilibrium was analyzed for each single-nucleotide polymorphism (SNP) with SNPStats (http://bioinfo.iconcologia.net/snpstats/start.htm). Fisher's exact test was applied when expected frequency was lower than 5. The *P* value was multiplied by the number of alleles observed for corrected *P* value (*P*c) to account for multiple comparisons performed. A *P*c value < 0.05 was considered statistically significant. Haldane's formula correction was used when critical entries were equal to zero.

## 3. Results

Allele frequencies of 15 SNPs in AITD and controls are in Table 3. For overall AITD, the allele frequencies of total TLR genes were not significantly different with controls. When AITD were categorized by the disease subgroup, GD showed higher frequencies of *TLR4 rs1927911* C allele (OR = 1.56; 95% CI, 1.0–2.4, *P* = 0.046) than those by the control group (Table 3).

When GD was categorized by TAO, the TAO group showed lower frequency of the *TLR4 rs1927911* CT genotype (OR = 0.4; 95% CI, 0.18–1.00, *P* = 0.047) and the non-TAO group showed a higher frequency of the *rs1927911* CC genotype (OR = 2.31; 95% CI, 1.07–4.99, *P* = 0.029) than the control group. Between the non-TAO and TAO groups, the frequency of the *TLR9 rs187084* CC genotype (OR = 5.52; 95% CI, 1.06–28.7, *P* = 0.028) in the TAO group was higher than that in the non-TAO group (Table 4).

When GD was categorized by sex, GD females showed a higher frequency of the *TLR4 rs10759932* T allele (OR = 2.06; 95% CI, 1.13–3.74, *P* = 0.015, *P*c = 0.03), *rs1927911* CC genotype (OR = 2.36; 95% CI, 1.23–4.52, *P* = 0.008, *P*c = 0.026), and *rs1927911* C allele (OR = 1.96; 95% CI, 1.18–3.26, *P* = 0.009, *P*c = 0.018) than controls. GD males showed a higher frequency of the *TLR4 rs10759932* CC genotype (OR = 4.34; 95% CI, 1.21–15.60, *P* = 0.031) and *rs1927911* TT genotype (OR = 3.61; 95% CI, 1.1–11.87, *P* = 0.033) and lower frequency of the *rs1927911* CT genotype (OR = 0.18; 95% CI, 0.04–0.82, *P* = 0.011, *P*c = 0.032) than controls (Table 5).

**Figure 1:**
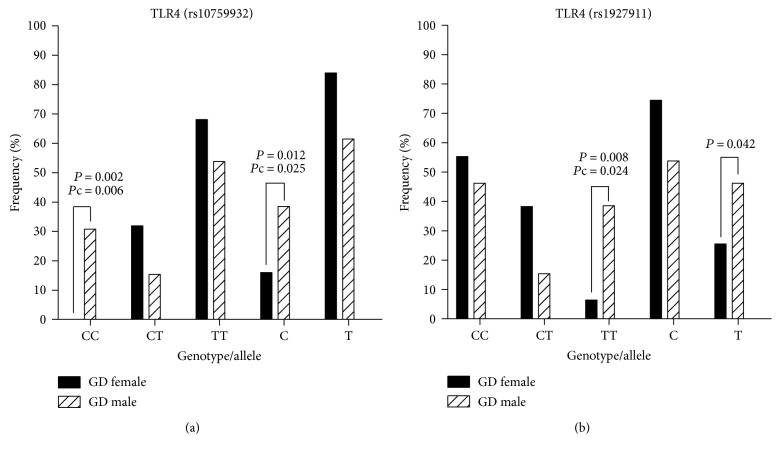
Analysis of *TLR*4 gene polymorphism between female and male in Graves' disease (GD); the frequency of the *TLR4 rs10759932* CC genotype (OR = 24; 95% CI, 2.52–228.3, *P* = 0.002, *P*c = 0.006), C allele (OR = 3.29; 95% CI, 1.26–8.63, *P* = 0.012, *P*c = 0.025), and *TLR4 rs1927911* TT genotype (OR = 9.17; 95% CI, 1.82–46.20, *P* = 0.008, *P*c = 0.024); and T allele (OR = 2.5; 95% CI, 1.02–6.15, *P* = 0.042) in GD female was lower than that in GD male.

Between females and males in GD, the frequency of the *TLR4 rs10759932* CC genotype (OR = 24; 95% CI, 2.52–228.3, *P* = 0.002, *P*c = 0.006), C allele (OR = 3.29; 95% CI, 1.26–8.63, *P* = 0.012, *P*c = 0.025), and *TLR4 rs1927911* TT genotype (OR = 9.17; 95% CI, 1.82–46.20, *P* = 0.008, *P*c = 0.024), T allele (OR = 2.5; 95% CI, 1.02–6.15, *P* = 0.042) in GD female was lower than that in GD male (Figure 1).

## 4. Discussions

In the present study, we found significant differences in genotype frequencies of *TLR4* gene polymorphisms in patients with GD. For GD, the *TLR4 rs1927911* C allele showed a disease-susceptible gene. The *TLR4* gene, located at chromosome 9q32-q33, recognizes lipopolysaccharides (LPS) of gram-negative bacteria and fusion proteins and envelope proteins of viruses as ligands. It is surface expressed and recognizes extracellular ligands and microorganisms [20]. Previous studies have reported that the disease associations of *TLR4* with chronic inflammatory disease include atherosclerosis, asthma, and rheumatoid arthritis [21]. It has been discovered that there is an association of *TLR4 rs1927911* SNP with childhood asthma [22] and disease activity of rheumatoid arthritis [23] and type 2 diabetes mellitus [24]. Nicola et al. reported that all components of the LPS receptor complex are expressed on thyrocyte, and they also detected that thyroid cells recognize and respond to LPS using Fisher rat thyroid cell line-5 cells [8]. Combined with aforementioned evidence, we can suggest that *TLR4* SNP could affect the pathogenesis of GD.

When GD was analyzed by TAO and compared with the control group, the *TLR4 rs1927911* CC and CT showed a significant protective genotype for TAO. *TLR4* signals via both the MyD88-independent pathway and MyD88-dependent pathways lead to robust IL-12 production, secretion of type I IFNs, and a string of Th1-type cellular and humoral immune responses [25]. We reported that the *IL-12* gene could be involved in the pathogenesis of TAO in Korean children [26]. The present study has a similar purpose in terms of investigating the immunogenetics of Korean AITD adolescents, but the specific target genes are clearly different with our previous research report. Dysregulation of the *TLR4* signaling owing to SNPs may alter the ligand binding and balance between pro- and anti-inflammatory cytokines, thereby modulating the risk of chronic inflammation [27]. Some reports suggest *TLR4 rs4986790* SNP and differences in LPS responsiveness in humans [28] and attenuated receptor signaling and diminished the inflammatory response to gram-negative pathogens [29]. The associations between *TLR4 rs10759931* SNP and *TLR4* expression in colon cancer tissues [30], *TLR2*-196 to *TLR2*-174del SNP, and *TLR2* mRNA expression have been reported [31]. Variants in *TLR2* and *TLR4* were associated with monocyte receptor levels of *TLR2* and *TLR4*, respectively, in a biracial cohort of adults [32]. Based on previous evidence, we may propose that *TLR4* SNPs are associated with TAO because of a process in the activation of immune cell signaling through cytokine production. However, studies on the possible consequences of *TLR4* SNPs in the function of the receptor are lacking and further large scale, well-designed, comprehensive studies are necessary in the future.

Between TAO and non-TAO, the frequency of the *TLR9 rs187084* CC genotype in non-TAO is lower than that in TAO. *TLR9* gene, located chromosome 3p21.3, recognizes CpG-containing DNA and DNA sugar backbone as ligand and is expressed in immune cells in intracellular endosomal compartments. Disease associations of *TLR9* with SLE, type 1 DM, multiple sclerosis, inflammatory bowel disease, and rheumatoid arthritis have been reported [21]. In 2010, Liao et al. reported that the frequency of the *TLR9 rs187084* CC genotype in non-TAO (6.25%) was lower than that in TAO (7.4%) in Taiwanese males [12]. Therefore, the results of our study are similar to those of the study conducted in Taiwanese patients. Based on previous evidence, we suspected that *TLR9 rs187084* SNPs are associated with TAO.

AITD prevalence is female predominant and the ratio of female and male is approximately 7 : 3 [33]. Females generate more robust humoral and cell-mediated immune responses after following antigenic challenge than males. These elevated immune responses in females may underlie the higher incidence of an array of disorders thought to be autoimmune in origin. However, the biology of sexual dimorphism in autoimmune disease is not clearly understood. Previous reports suggest that X chromosome inactivation is an important contributor to the increased risk of females for developing AITD [34–36]. Sexual dimorphism in the expression of *TLR4* for bacterial LPS in murine macrophages has been reported, and *TLR4* might contribute to the greater susceptibility of males to bacteria sepsis [10]. In this study, we observed that GD females showed a higher frequency of the *TLR4 rs10759932* T allele, *rs1927911* CC genotype, and *rs192791* C allele than controls. GD males showed a higher frequency of the *TLR4 rs10759932* CC and *rs1927911* TT genotypes and a lower frequency of the *rs1927911* CT genotype than controls. Between GD male and GD female, the frequency of the *TLR4 rs10759932* CC genotype, C allele, *TLR4 rs1927911* TT genotype, and T allele was lower. *TLR4 rs10759932* SNP has been reported to be associated with childhood asthma [37] and psoriasis vulgaris [38]. These results might suggest that *TLR4* polymorphisms might influence the female predominance of GD and act as evidence explaining AITD pathogenesis.

*TLR3* overexpression in thyrocytes from patients with HD has been reported, but not in normal thyrocytes or patients with GD. *TLR3* overexpression induces an innate immune response in thyrocytes, which may be important in HD pathogenesis and in immune cell infiltrates [7]. Several *TLR1*, *2*, and *6* polymorphisms have been described with functional and genetic association studies including asthma, rheumatoid arthritis, and inflammatory bowel disease [6, 21]. In this study, we tried to investigate the association between *TLR1 rs4833095*, *TLR2 rs4696480*, *rs1898830*, *rs7656411*, *TLR3 rs3775291*, *rs3775296*, *TLR5 rs5744168*, *TLR6 rs5743810*, *rs2381289*, and AITD. However, there were no significant differences in genotype frequencies of *TLR1*, *2*, *3*, *5*, and *6* between AITD and controls.

There are some limitations in this study. First, the control group in this research consisted mainly of both teaching and nonteaching staff and students from the College of Medicine at the Catholic University of Korea. The entire population of healthy control patients (adult population) is different from that of patients under analysis (pediatric population). Because there is no report that the distribution of HLA genotypes varies with age in a single race, the healthy controls of the adult population were used in this study despite the difference from that of patients under analysis (pediatric population). Furthermore, it is difficult to obtain enough pediatric control for ethical reason. Second, this study has a small number of cases and controls. Although there was limitation in getting a sufficient amount of samples, especially, pediatric AITD patient's samples, we were able to demonstrate the significant genetic associations of *HLA*, *MICA*, *TLR10*, and cytokine with AITD in pediatric patients which was conducted with a similar number of patient's samples as in this study [11, 13, 26, 39].

In conclusion, we suggest that *TLR4* SNP may be involved in the pathogenesis of GD and *TLR9* SNP could affect the pathogenesis of TAO. We also observed sexual dimorphism in the *TLR4* gene in GD. Our data could be also used as baseline data for understanding the pathophysiology of AITD.

## Figures and Tables

**Table 1:** Characteristics of 104 autoimmune thyroid disease patients.

Characteristics	
Sex (F/M)	86/18
Age (years) at diagnosis	11.3 ± 3.2
Age (years) at enrollment	13.2 ± 3.5
HD/GD	44/60
HD condition at diagnosis	
Euthyroid state	9 (20.5%)
Subclinical hypothyroid state	6 (13.6%)
Overt hypothyroid state	23 (52.3%)
Hyperthyroid state	6 (13.6%)
HD patients on T4 replacement	25 (56.8%)
Class of TAO	
0 ~ 1 no sign ~ only sign	75
2 soft tissue involvement	7
3 proptosis	19
4 extraocular muscle involvement	3
5 corneal involvement	0
6 sight loss	0

AITD: autoimmune thyroid diseases; HD: Hashimoto's disease; GD: Graves' disease; TAO: thyroid-associated ophthalmopathy.

**Table 2:** Primer sequences for each single-nucleotide polymorphism.

Gene	SNP	Direction	Primer sequence (5′ → 3′)
*TLR1*	*rs4833095*	F	GCCAAACCAGCTGGAGGATCC
	(*+742*)	R	TGGGGAACACAATGTGCAGACTC

*TLR2*	*rs4696480*	F	CAAGATTGAAGGGCTGCATCTGG
	(*−16934*)	R	CCACCTCTCAGCTCGCAGTGAG
	*rs1898830*	F	GAAGAGTGACGAAAAATGAATGAGCA
	(*intron1*)	R	GATGAACCTCTGGCAAGACAATAAAAG
	*rs7656411*	F	GCCTGCCCTTTTTCCCCTTC
	(*3*′*UTR*)	R	TTAAGCTGGGAACCATGTGAAAGG

*TLR3*	*rs3775291*	F	TGGCCCAACCAAGAGAAAGCA
	(*c.1243*)	R	TGGGGAGTGAGGCAAGGGAA
	*rs3775296*	F	CCAATGCATTTGAAAGCCATCTG
	(*−7*)	R	CCTTTTGCCCTTTGGGATGC

*TLR4*	*rs11536889*	F	TGGGCAATGCTCCTTGACCAC
	(*3*′*UTR*)	R	GGACAATCAGGATGTCATCAGGGA
	*rs10759932*	F	CACTTGCTACTTTCCAGACACTGTCCT
	(*−1607*)	R	TGAGAACTCCTGTACACCATTTGTGG
	*rs1927911*	F	TGGCCCAGATTTTGACAACTGC
	(*intron1*)	R	CATGGATTCCCATGGTGGAACC

*TLR5*	*rs5744168*	F	TCTGGGGGAACTTTACAGTTCGAA
	(*+1174*)	R	TGCCAAAGATCAACCTTACAGCG

*TLR6*	*rs5743810*	F	CCCTTCACCTTGTTTTTCACCCA
	(*+745*)	R	CTTGGAAATGCCTGGTCAGAGTCT
	*rs2381289*	F	CTGCAAGGAAGGCCAAGCAGA
	(*3*′*UTR*)	R	TGAAGCCCTCGCTTTCTGGACT

*TLR9*	*rs352140*	F	CGCTGACCGGTCTGCAGGT
	(*+2848*)	R	ACTGGAGGCCCTGGACCTCA
	*rs187084*	F	ACTGGATCCTGGGGATGCAGA
	(*−1486*)	R	AGCTGACATTCCAGCAGGGGA
	*rs352162*	F	TCTCCTGAATCTCCAGCCCCA
	(*3*′*UTR*)	R	TGGCAATCCCAAGACAGGCA

SNP: single-nucleotide polymorphism; *TLR*: *Toll-like receptor*.

**Table 3:** Allele frequencies of *Toll-like receptor* polymorphism in controls and patients with thyroid disease.

	SNP alleles	Normal	AITD	GD	HD
		2*n* = 366 (%)	2*n* = 208 (%)	2*n* = 120 (%)	2*n* = 88 (%)
*TLR1* (*+742*)	C	235 (64.2)	126 (60.6)	74 (61.7)	52 (59.1)
*rs4833095*	T	131 (35.8)	82 (39.4)	46 (38.3)	36 (40.9)
*TLR2* (*−16934*)	A	187 (51.1)	115 (55.3)	65 (54.2)	50 (56.8)
*rs4696480*	T	179 (48.9)	93 (44.7)	55 (45.8)	38 (43.2)
*TLR2* (*intron1*)	A	185 (50.5)	112 (53.8)	66 (55.0)	46 (52.3)
*rs1898830*	G	181 (49.5)	96 (46.2)	54 (45.0)	42 (47.7)
*TLR2* (*3*′*UTR*)	G	180 (49.2)	116 (55.8)	70 (58.3)	46 (52.3)
*rs7656411*	T	186 (50.8)	92 (44.2)	50 (41.7)	42 (47.7)
*TLR3* (*c.1243*)	A	111 (30.3)	59 (28.4)	31 (25.8)	28 (31.8)
*rs3775291*	G	255 (69.7)	149 (71.6)	89 (74.2)	60 (68.2)
*TLR3* (*−7*)	A	80 (21.9)	49 (23.6)	24 (20.0)	25 (28.4)
*rs3775296*	C	286 (78.1)	159 (76.4)	96 (80.0)	63 (71.6)
*TLR4* (*3*′*UTR*)	C	83 (22.7)	49 (23.6)	31 (25.8)	18 (20.5)
*rs11536889*	G	283 (77.3)	159 (76.4)	89 (74.2)	70 (79.5)
*TLR4* (*−1607*)	C	103 (28.1)	47 (22.6)	25 (20.8)	22 (25.0)
*rs10759932*	T	263 (71.9)	161 (77.4)	95 (79.2)	66 (75.0)
*TLR4* (*intron1*)	C	219 (59.8)	135 (64.9)	84 (70.0)^a^	51 (58.0)
*rs1927911*	T	147 (40.2)	73 (35.1)	36 (30.0)	37 (42.0)
*CC-TLR5* (*+1174*)	C	362 (98.9)	205 (98.6)	118 (98.3)	87 (98.9)
*rs5744168*	T	4 (1.1)	3 (1.4)	2 (1.7)	1 (1.1)
*CC-TLR6* (*+745*)	C	366 (100.0)	208 (100.0)	120 (100.0)	88 (100.0)
*rs5743810*	G	0 (0.0)	0 (0.0)	0 (0.0)	0 (0.0)
*TLR6* (*3*′*UTR*)	C	180 (49.2)	117 (56.3)	66 (55.0)	51 (58.0)
*rs2381289*	T	186 (50.8)	91 (43.7)	54 (45.0)	37 (42.0)
*TLR9* (*+2848*)	A	150 (41.0)	89 (42.8)	50 (41.7)	39 (44.3)
*rs352140*	G	216 (59.0)	119 (57.2)	70 (58.3)	49 (55.7)
*TLR9* (*−1486*)	C	152 (41.5)	91 (43.8)	51 (42.5)	40 (45.5)
*rs187084*	T	214 (58.5)	117 (56.2)	69 (57.5)	48 (54.5)
*TLR9* (*3*′*UTR*)	C	153 (41.8)	90 (43.3)	50 (41.7)	40 (45.5)
*rs352162*	T	213 (58.2)	118 (56.7)	70 (58.3)	48 (54.5)

SNP: single-nucleotide polymorphism; AITD: autoimmune thyroid diseases; HD: Hashimoto's disease; GD: Graves' disease; *TLR*: *Toll-like receptor*. Controls versus GD: ^a^OR = 1.56 (1.0–2.4), *P* = 0.046.

**Table 4:** Genotype frequencies of *TLR4* genes between controls and patients with or without thyroid-associated ophthalmopathy in Graves' disease.

		Normal	GD TAO	GD non-TAO
		*n* = 183 (%)	*n* = 29 (%)	*n* = 31 (%)
*TLR4 (intron1)*	CC	63 (34.4)	15 (51.7)	17 (54.8)^b^
*rs1927911*	CT	93 (50.8)	9 (31.0)^a^	11 (35.5)
	TT	27 (14.8)	5 (17.2)	3 (9.7)
	C	219 (59.8)	39 (67.2)	45 (72.6)
	T	147 (40.2)	19 (32.8)	17 (27.4)

*TLR9 (−1486)*	CC	35 (19.1)	8 (27.6)	2 (6.5)^c^
*rs187084*	CT	82 (44.8)	12 (41.4)	19 (61.3)
	TT	66 (36.1)	9 (31.0)	10 (32.3)
	C	152 (41.5)	28 (48.3)	23 (37.1)
	T	214 (58.5)	30 (51.7)	39 (62.9)

AITD: autoimmune thyroid diseases; GD: Graves' disease; TAO: thyroid-associated ophthalmopathy. Controls versus TAO: ^a^OR = 0.4 (0.18–1.00), *P* = 0.047; controls versus non-TAO: ^b^OR = 2.31 (1.07–4.99), *P* = 0.029; TAO versus non-TAO: ^c^OR = 5.52 (1.06–28.7), *P* = 0.028.

**Table 5:** Genotype frequencies of *TLR4* genes between controls and female or male in GD.

		Normal	GD female	GD male
		*n* = 183 (%)	*n* = 47 (%)	*n* = 13 (%)
*TLR4 (−1607)*	CC	17 (9.3)	0 (0.0)	4 (30.8)^d^
*rs10759932*	CT	69 (37.7)	15 (31.9)	2 (15.4)
	TT	97 (53.0)	32 (68.1)	7 (53.8)
	C	103 (28.1)	15 (16.0)	10 (38.5)
	T	263 (71.9)	79 (84.0)^a^	16 (61.5)

*TLR4 (intron1)*	CC	63 (34.4)	26 (55.3)^b^	6 (46.2)
*rs1927911*	CT	93 (50.8)	18 (38.3)	2 (15.4)^e^
	TT	27 (14.8)	3 (6.4)	5 (38.5)^f^
	C	219 (59.8)	70 (74.5)^c^	14 (53.8)
	T	147 (40.2)	24 (25.5)	12 (46.2)

AITD: autoimmune thyroid diseases; GD: Graves' disease. Controls versus GD female: ^a^OR = 2.06 (1.13–3.74), *P* = 0.015, *P*c = 0.03; ^b^OR = 2.36 (1.23–4.52), *P* = 0.008, *P*c = 0.026; ^c^OR = 1.96 (1.18–3.26), *P* = 0.009, *P*c = 0.018; controls versus GD male: ^d^OR = 4.34 (1.21–15.60), *P* = 0.031; ^e^OR = 0.18 (0.04–0.82), *P* = 0.011, *P*c = 0.032; ^f^OR = 3.61 (1.1–11.87), *P* = 0.033.
